# Summary Concerning 16 Years of Research Activities in the Department of Pediatrics at Juntendo University

**DOI:** 10.14789/ejmj.JMJ24-0029-P

**Published:** 2025-01-30

**Authors:** TOSHIAKI SHIMIZU

**Affiliations:** 1 Department of Pediatrics and Adolescent Medicine, Juntendo University Graduate School of Medicine, Tokyo, Japan; 1 Department of Pediatrics and Adolescent Medicine, Juntendo University Graduate School of Medicine, Tokyo, Japan

**Keywords:** research activity, inflammatory bowel disease, omega-3 fatty acids, epigenetics, transition

## Abstract

I described mainly about my research activities in the 16 years and 8 months since I became professor of pediatrics at Juntendo University. Since the fulfillment of research activities has a great influence on clinical activities, and sufficient research cannot be conducted without enhanced educational activities, I have tried to maintain a good balance between clinical practice, research, and education. Research activities in our department often depend on the research abilities of graduate students, but the leadership skills of the professors and associate and assistant professors who supervise the research are also important. First, I talked about the main research of the 12 research groups in our department. In addition, my specialty is pediatric gastroenterology and nutrition, and it is no exaggeration to say that Juntendo Pediatrics is one of the leaders in the field of pediatric gastroenterology and nutrition. I also provided a detailed explanation of research on pediatric inflammatory bowel disease (IBD), which is currently the most important research topic in our department. I hope that this environment will continue to foster the research mindset of young people in a natural way.

## Introduction

I have just completed 16 years and eight months of works as the chair and professor of the Department of Pediatrics, Juntendo University Faculty of Medicine. I am extremely happy to have had this experience and would like to express my gratitude to everyone involved. Many things have happened during this time, but we could maintain a good balance between clinical, research and educational activities with keywords such as “nurture,” “collaboration,” “development” and “succession.”

Methods for evaluating the research strength of universities and departments include the number of English-language papers, their impact factors and citation indexes, the number of medical doctorates awarded, and the number of competitive external funding received, and Juntendo Pediatrics is among the top in all these areas, both within the university and among pediatric departments at university hospitals nationwide. Twelve research groups are conducting research activities, primarily with graduate students, and over the past 16 years, 115 people have earned degrees (104 A-papers and 11 B- papers). Many members of the department have also received scientific research grants from the Ministry of Education, Culture, Sports, Science and Technology and the Ministry of Health, Labor and Welfare, as well as Juntendo's project research grants.

It is thanks to the many young people who have come to Juntendo University's Pediatrics Department that we have been able to achieve such great results in research. In fact, 220 people have joined our department in the last 16 years, and I believe that the main reason for this is that our department's enthusiasm for education has been conveyed to them. In fact, this enthusiasm for education has also led to research guidance for graduate students, and it can be said that the enhancement of our educational capabilities has greatly contributed to the development of our department in both clinical and research aspects.

Here, I would like to mainly explain the research activities by graduate students in our department over the 16 years since I became the professor.

## Activities of 12 research groups

Normally, even university hospitals do not have clinical groups that can handle the entire range of pediatric clinical fields, but our department's major selling point is that we have clinical groups that can handle 12 clinical fields. Accordingly, we also have 12 research groups, each of which is engaged in research activities in a different field, which is another strength of our department. Table 1 shows the 12 research groups in our department. Not only do we receive guidance from many of Juntendo's department and laboratories, but we also actively conduct collaborative research with the external facilities. Although there are some differences between groups, as mentioned above, a total of 115 people from all groups have earned their degrees over the past 16 years. Due to space limitations, I would like to introduce some of the research that has been conducted by graduate students in some of these groups.

**Table 1:** 12 research groups and the number of degree recipients in each group

Research groups	Number of degree recipient
Gastroenterology	20
Cardiology	18
Allergology	11
Hematology・Oncology	11
Nephrology	10
Developmental psychology	8
Infectious diseases	8
Liver・Metabolic diseases	7
Neonatology	6
Neurology	6
Endocrinology	5
Nutrition	5

**Figure 1:**
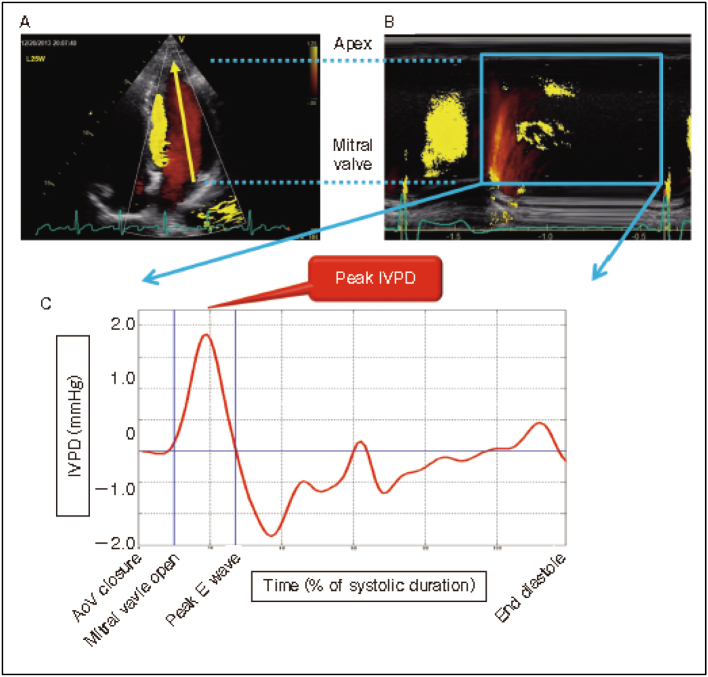
Evaluation of cardiac function using Speckle Tracking Imaging (Matsui et al.^1)^ 2000) Measurement of Intraventricular Pressure Difference (IVPD)

First, there is the cardiovascular group, which has the second largest number of doctoral degree holders after the gastroenterological group. Led by Professor Ken Takahashi, with Associate Professor Masahiko Kishiro, Hideo Fukunaga, and Kotoko Matsui as the main instructors, they have published doctoral dissertations on the evaluation of cardiac function in various childhood-onset diseases using Speckle Tracking Imaging (Figure 1)^1)^. As for various diseases, they have published papers on heart diseases such as tetralogy of Fallot^2, 3)^, WPW syndrome^4)^, as well as patients with hematological malignancies^5, 6)^, type 1 diabetes^7)^, and inflammatory bowel disease^8)^.

The nephrology group is also actively engaged in research activities both laboratories at Juntendo and external facilities. Led by Professor Yoshiyuki Otomo, and under the research guidance of senior Associate Professor Naoto Nishizaki, and Associate Professor Amane Endo and Mayu Nakagawa, the research evaluates renal function in juvenile disease models and examines prevention and treatment of disease progression. Doctoral dissertations have been published on the effects of renal dysfunction biomarkers in cyclosporine-induced nephropathy rats^9)^, the usefulness of mizoribine and angiotensin receptor blockers (ARBs)^10)^, the effectiveness of mizoribine, direct renin inhibitors^11)^, and hydrogen peroxide on fibrosis^12)^ in unilateral ureteral obstruction rats, renal dysfunction biomarkers in intrauterine growth restriction rats^13)^, and renal hypoplasia in a retinopathy model^14)^.

**Figure 2:**
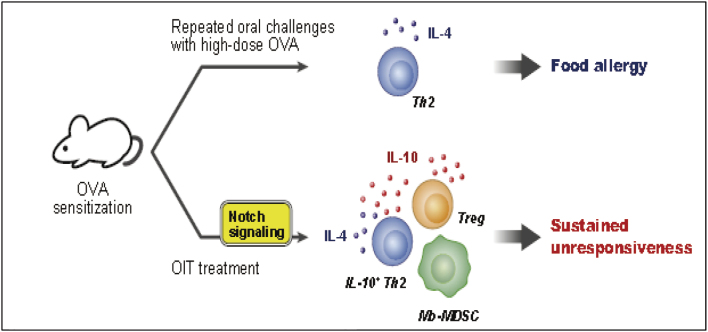
Research using food allergy murine model (Yamada et al.^17)^ 2023) The effects of various factors on resensitization were examined using mice sensitized with ovalbumin (OVA).

The allergy group is conducting high-level research using gut immunity and food allergy model mice (Figure 2) under the guidance of Visiting Professor Yoshikazu Otsuka, Senior Associate Professor Takahiro Kudo, Associate Professor Yosuke Baba, and Assistant Professor Eisuke Inage, as well as Professor Jiro Kitaura and others at the Atopy Research Center. Food allergies are a disease that often causes problems in actual clinical practice in children, and research leading to elucidation of the disease's pathophysiology and therapeutic intervention is being conducted and published^15-17)^.

**Figure 3:**
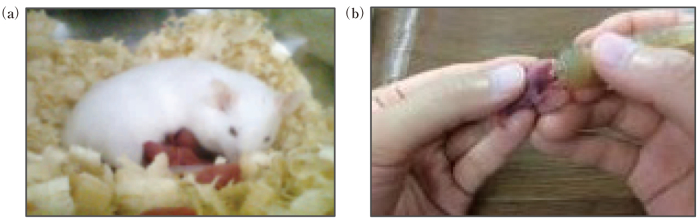
Immunological benefits of breast milk using artificial feeding (Sakaguchi et al.^24)^ 2018) Immediately after birth, rats were hand-fed with artificial feeding and immunological differences between breast-fed (a) and artificially fed (b) rats were examined.

The neonatal and nutrition group is my area of expertise, and is currently investigating the effects of perinatal nutrition on the future health of infants, led by Professor Masato Kantake, Senior Associate Professor Hiromichi Shoji and Naoto Nishizaki. Using a low-birth weight rat model, we are investigating the effects of IGF-1 on growth^18)^, insulin resistance in skeletal muscle^19)^, autophagy in the liver and skeletal muscle^20)^, and gut microbiota^21)^. We are also investigating the protective effect of DHA administration to pregnant rats on the brain of infants^22, 23)^, and the immunological benefits of breast milk using artificial feeding (Figure 3)^24)^. Regarding pediatric nutrition, various social activities are being carried out in response to requests from various government ministries and agencies and the Japan Pediatric Society, as shown in Table 2.

**Table 2:** Various social activities in the field of pediatric nutrition

1. Establishment of the "2020 Dietary Reference Intakes for Japanese" (Ministry of Health, Labor and Welfare)
2. Editing of the "Practical Guide for Breastfeeding and Weaning Support Guide" (Ministry of Health, Labor and Welfare)
3. Implementation of an infant nutrition survey (Ministry of Health, Labor and Welfare)
4. Study group on school lunch issues (Ministry of Education, Culture, Sports, Science and Technology)
5. Study group on the composition of liquid milk (Consumer Affairs Agency)
6. Fish promotion activities (Ministry of Agriculture, Forestry and Fisheries)
7. Promotion of nutrition education activities for children (Ministry of Agriculture, Forestry and Fisheries)
9. Addressing the issue of skipping breakfast (Japan Pediatric Society)
10. Measures to prevent biotin deficiency in special milk (Japan Pediatric Society)
11. Establishment of infant nutrition guidelines in Vietnam (50th anniversary of Japan-Vietnam diplomatic relations project)

## Pediatric gastroenterology as my main field

Juntendo University is a recognized leader in the field of pediatric nutrition and gastroenterology in Japan, and I have been working as the president to make the Japanese Society for Pediatric Gastroenterology, Hepatology and Nutrition, a division of the Japanese Pediatric Society, even more attractive. Therefore, our department has conducted a lot of research on inflammatory bowel disease (IBD), irritable bowel syndrome^25, 26)^, *Helicobacter pylori* infection^27, 28)^, acute and chronic pancreatitis^29, 30)^, and other subjects. We have been actively researching IBD in recent years, and I will discuss my research on IBD below.

**Figure 4:**
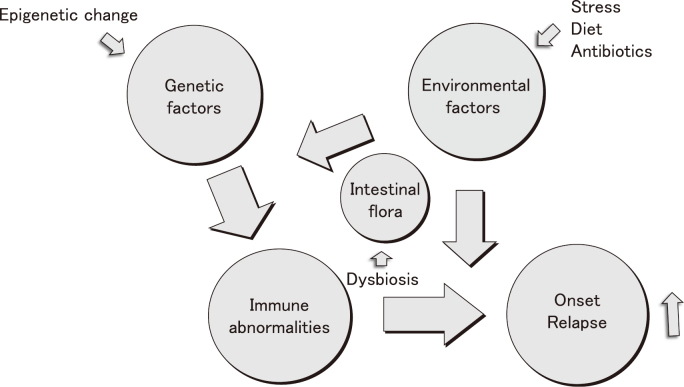
Causes of inflammatory bowel disease (Shimizu^31)^ 2023) A decrease in intake of ω-3 fatty acids and epigenetic changes are also thought to be one of the causes of the increase in IBD patients.

In the past, IBD was considered a rare disease in Japan, but now Japan is the rather common country in the world, and it is the most registered disease among the country's designated intractable diseases. The number of UC and CD patients has also increased significantly in children and is now two to three times as many as 20 years ago. There is still no clear answer to the question of why the number of IBD patients is increasing so much in Japan, but it seems important to consider the etiology. It is believed that environmental factors act on genetic predisposition, causing disorders of the intestinal flora and immune abnormalities in the digestive mucosa, leading to the onset or repeated relapse or worsening of IBD. Therefore, it is speculated that the recent increase in IBD patients is probably due to environmental factors such as stress and increased use of antibiotics, changes in living environment and diet, problems with genetic predisposition due to epigenetic changes, and even changes in the intestinal flora due to dysbiosis (Figure 4)^31)^.

## Increase in IBD patients and ω-3 fatty acids.

**Figure 5:**
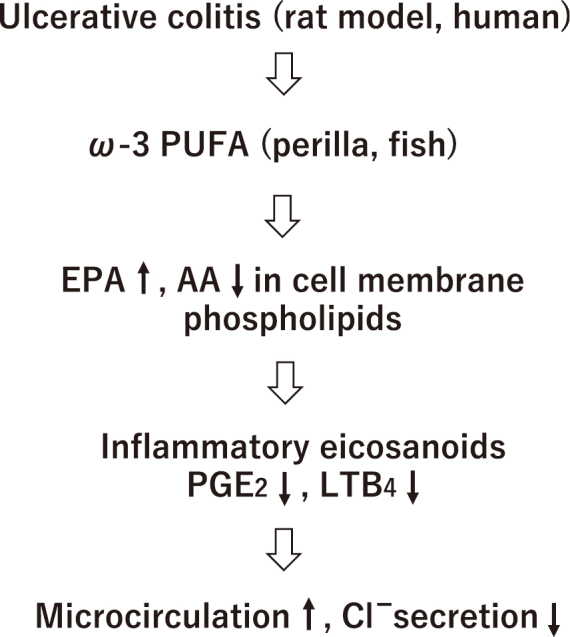
Pathogenesis of IBD and ω-3 fatty acids By administering ω-3 polyunsaturated fatty acids (ω-3PUFA) to IBD, eicosapentaenoic acid (EPA) increases and arachidonic acid (AA) decreases, suppressing the production of inflammatory eicosanoids biosynthesized from arachidonic acid and improving clinical symptoms.

At first, we will show the relationship between ω-3 fatty acid intake and the results of research in our department that may explain the recent increase in the number of IBD patients mentioned above. We first created a UC model in young rats using dextran sulfate sodium (DSS) and used this UC model to evaluate the usefulness of ω-3 fatty acids using a laser Doppler blood flow meter and an Ussing chamber^32, 33)^. In addition, as a clinical study, we administered an eicosapentaenoic acid (EPA) preparation, an ω-3 fatty acid, to children with UC and examined its remission maintenance effect^34)^. The results confirmed that in rat models and human UC, administration of perilla oil and fish oil, which are ω-3 polyunsaturated fatty acids, increased the EPA concentration in cell membrane phospholipids and decreased the arachidonic acid concentration. As a result, the production of inflammatory eicosanoids PGE_2_ and LTB_4_, which are biosynthesized from arachidonic acid, was suppressed, and we believe that the clinical symptoms of IBD improved, and remission was maintained through improved microcirculation and suppression of chloride ion secretion (Figure 5). Therefore, it is possible that a deficiency of ω-3 fatty acids leads to increased production of inflammatory eicosanoids and is involved in the onset of IBD. In fact, the intake of seafood, which is the largest source of ω-3 fatty acids, has been decreasing year by year, and according to the National Health and Nutrition Survey, the intake of seafood was higher than the intake of meat, which is rich in ω-6 fatty acids, but since around 2008, the intake of seafood has been lower than the intake of meat, and recently the intake of seafood is about 60% of the intake of meat. In other words, it is thought that the decrease in the intake of ω-3 fatty acids such as seafood in Japanese people has led to an increase in arachidonic acid and inflammatory eicosanoids, leading to an increase in IBD cases.

## Increase in IBD patients and epigenetic changes

**Figure 6:**
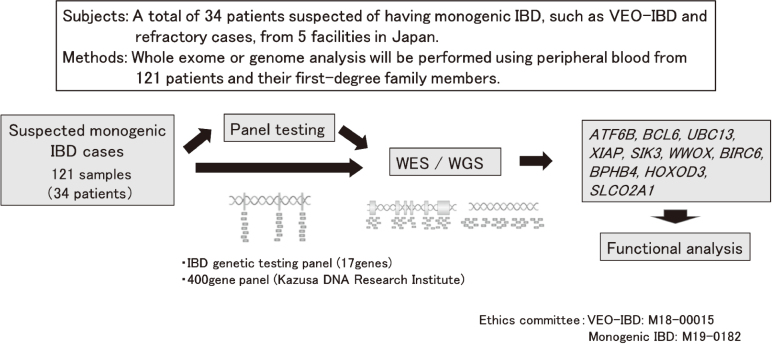
Identification of new causative genes in monogenic IBD and their functional analysis

Another research result from our department that may explain the recent increase in IBD cases is that epigenetic changes are involved in the onset of IBD. While genetic predisposition, environmental factors, and even intestinal flora and immune abnormalities are thought to be involved in the pathogenesis of inflammatory bowel disease, it has been discovered that in recent years, there are a significant number of cases of pediatric IBD caused by a single pathological gene, so-called monogenic IBD. In a nationwide survey conducted by our department, of 225 cases of VEO-IBD that developed before the age of 6, 26 cases (11.6%) were found to have monogenic IBD associated with primary immunodeficiencies such as chronic granulomatous disease, IL-10 receptor abnormality, A20/TNFAIP3 gene abnormality, and XIAP deficiency (Table 3)^35)^. The significance of diagnosing monogenic IBD is that it may be possible to cure monogenic IBD, which can be fatal, with appropriate treatment, and that identification of the causative gene and functional analysis of the expressed protein may lead to the development of targeted molecular drugs or the prediction of extraintestinal lesions and infections. In fact, we have experienced a case of IBD that began at a young age and was resistant to treatment, so we performed genetic testing, diagnosed it as XIAP deficiency or IL-10 receptor abnormality, and cured it by bone marrow transplantation^36)^. Currently, in our department, for cases suspected of monogenic IBD, we perform whole-exome or whole-genome analysis on cases that were negative in the IBD genetic testing panel that tests 17 genes, search for candidate genes, and perform functional analysis (Figure 6). Here, we introduce some of the research results that may explain the recent increase in IBD.

**Table 3:** Diagnosis of 225 patients with VEO-IBD

Diagnosis	No. of patients	（％）
Ulcerative colitis	12	56.0
Crohn’s disease	45	21.3
IBD unclassified	18	8.0
Behcet’s Disease	7	3.1
Immunodeficiency-associated enteritis	26	11.6
Chronic granulomatous disease	6	
Interleukin-10 receptor deficiency	4	
A20 haploinsufficiency	3	
X-linked lymphoproliferative disease type 2	3	
MHC class Ⅱ deficiency	1	
IKBA gene disorder	1	
MIRAGE syndrome	1	
Sever combined immunodeficiency disease	1	
Hoyeraal-Hreidarsson syndrome	1	
Coffin-Siris syndrome	1	
Wiskott-Aldrich syndrome	1	
IPEX syndrome	1	
Interleukin-2Rαdeficiency	1	
Undetected causative gene	1	

IBD, inflammatory bowel disease; VEO-IBD, very early-onset inflammatory bowel disease.

**Figure 7:**
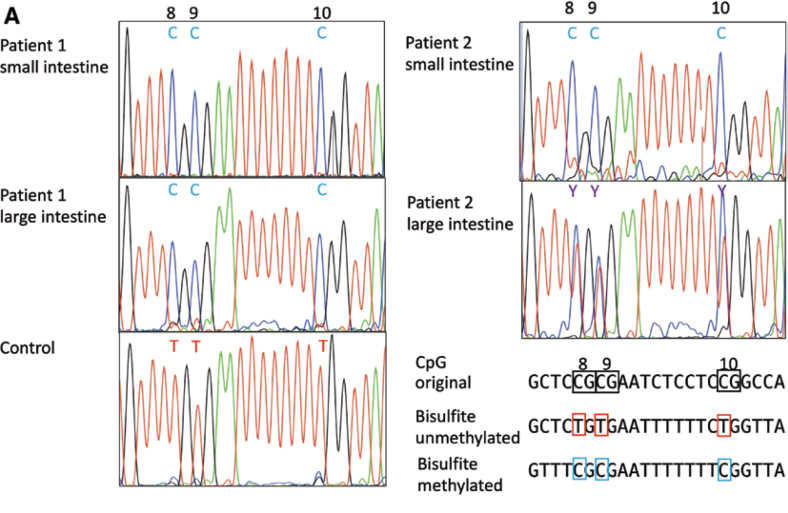
Bisulfite sequencing results (Ito et al.^37)^ 2023) Complete DNA methylation in the inflamed mucosa of both small and large intestines was observed in patient 1, however a highly methylated pattern and partial methylation were observed in the inflamed mucosa of the small intestine and noninflamed mucosa of the large intestine, respectively, in patient 2.

**Figure 8:**
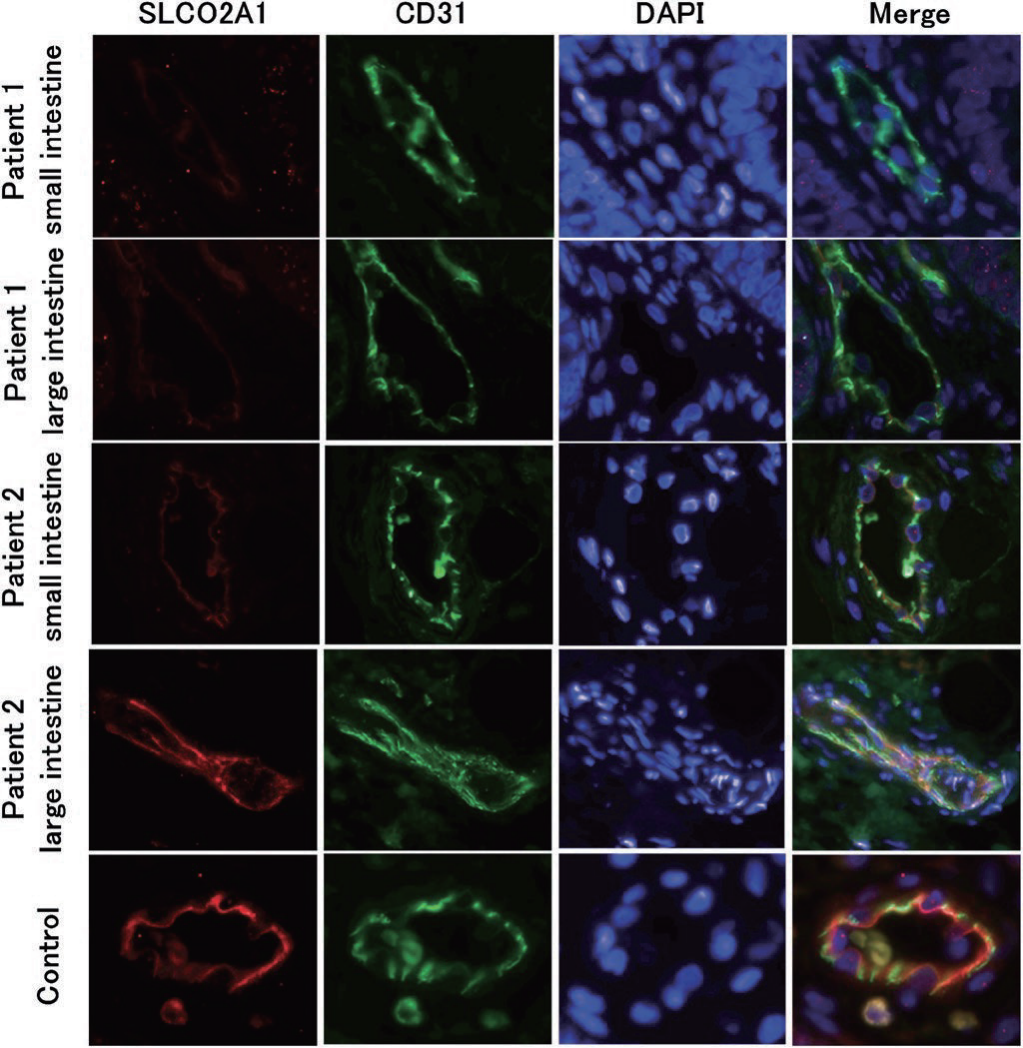
Immunofluorescence staining of bowel biopsy samples (Ito et al.^37)^ 2023) The protein expression of SLCO2A1was considerably lower in the vascular endothelial cells of inflamed mucosa in the small and large intestine in patient 1 and the small intestine in patient 2 than in the control samples, although the expression in the noninflamed colonic mucosa in patient 2 was almost comparable with that in the control samples.

Ito, et al.^37)^ reported two sisters, one who developed IBD at age 2 and the other who developed it at age 9. The younger sister had lesions in both the small and large intestines, whereas the older sister had lesions only in the small intestine^8)^. Genetic analysis revealed that both sisters were homozygous for the SLCO2A1 mutation, which causes nonspecific multiple small intestinal ulcers. We therefore considered the possibility that epigenetic changes caused by secondary influences such as intestinal bacteria had led to acquired inactivation of normal alleles and performed DNA methylation analysis of intestinal tissue. The results showed complete methylation in the small and large intestines of the younger sister, whereas only partial methylation was observed in the large intestine of the older sister, who had no lesions (Figure 7). Furthermore, the results of SLCO2A1 RNA expression and protein expression analysis (Figure 8), as well as the measurement of urinary PG metabolites, correlated with the degree of DNA methylation, suggesting that DNA methylation may be involved in the onset of IBD in these sisters, and that this epigenetic change may be involved in the recent increase in IBD patients.

## IBD-related papers involving our department

The number of papers involving pediatric IBD involving our department was four in the 10 years from 2000 to 2009, but the number has increased dramatically, to 18 in the next 10 years (2010 to 2019) and 27 in the last four years (2020 to 2023). Recent papers cover a wide range of topics, including VEO-IBD and monogenic IBD, accuracy of transperineal ultrasonography, effectiveness of molecular targeted drug therapy in multi-institutional collaborative studies, expression of cancer-related genes, transitional medical care, and COVID-19-related topics. Three of these papers are introduced here.

**Figure 9:**
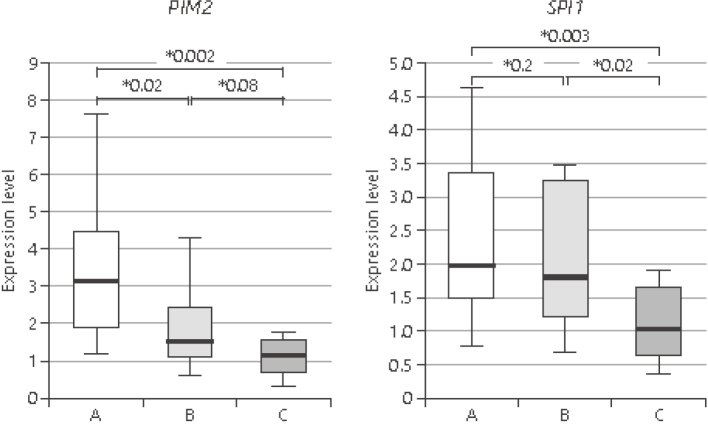
Real-time PCR analysis of *PIM2* and *SPI1* expressions in rectal mucosal biopsy specimens (Arai et al.^38)^ 2022) Expression levels of oncogenic *PIM2* and SPI1 were significantly higher in Group A (active UC) than in Group C (control). The expression level of *PIM2* decreased significantly between Group A and Group B (inactive UC), whereas the expression level of *SPI1* did not differ between these 2 UC groups.

**Figure 10:**
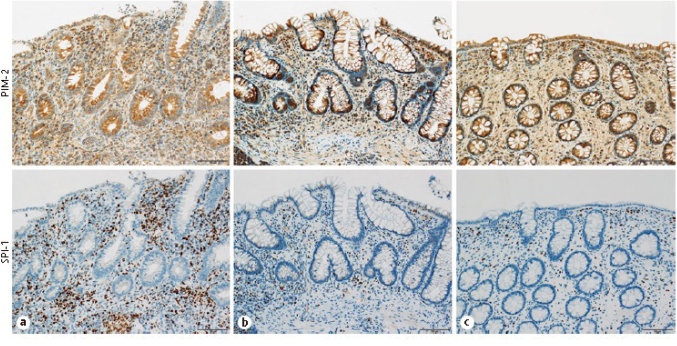
Immunohistochemistry staining of *PIM2* and *SPI1* expressions in rectal mucosal biopsy specimens (Arai et al.^38)^ 2022) Staining of target proteins by immunohistochemistry revealed the same expression patterns observed by real-time PCR.

Arai et al.^38)^, a graduate student in our department, analyzed the expression of cancer-related genes in the colonic mucosa of pediatric UC patients and examined the risk of developing colon cancer in the future. When real-time PCR was used to analyze cancer-related molecules whose expression was increased by the microarray method, the expression of PIM2 and SPI1 was significantly increased in the active group (p<0.05) (Figure 9), while the expression of TP53 and APC, which are known to be increased in adults, was significantly decreased (p<0.05). Immunohistochemical staining for those proteins supported the real-time PCR results (Figure 10), Expression levels of previously unreported cancer-related genes in adult UC patients were significantly higher in pediatric UC patients than in controls. Inflammation of the gastrointestinal mucosa increased the expression levels of cancer-related genes even in childhood-onset UC cases, suggesting the chronic inflammation from childhood may increase the risk colorectal cancer development.

**Figure 11:**
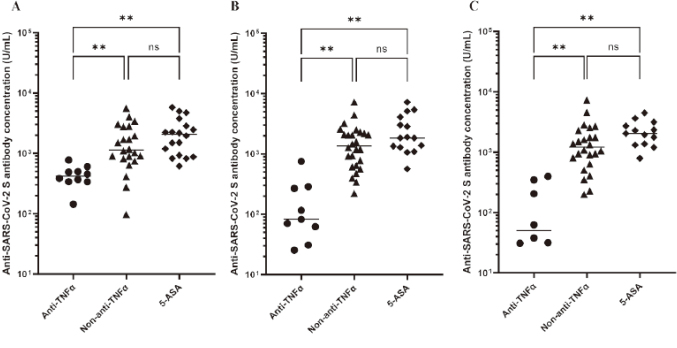
Anti-SARS-CoV-2 S antibody concentrations after the initial two-vaccine series (Kasiwagi et al.^39)^ 2022) The geometric mean of anti-SARS-CoV-2 S protein antibody concentrations following the second vaccination was significantly lower in patients treated with anti-TNFα than in those treated with non-anti-TNFα and 5-ASA at all measurement points (A: after 3-9 weeks, B: after 10-16 weeks, C; after 20-28 weeks).

**Figure 12:**
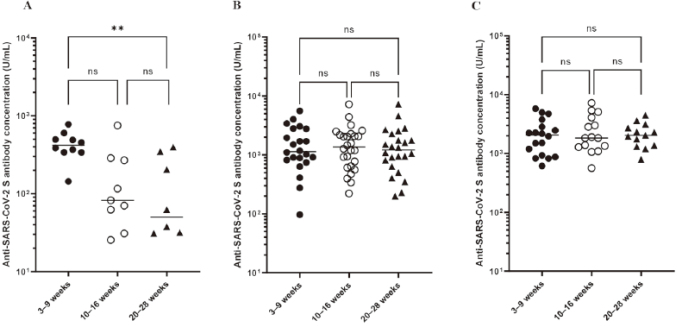
Anti-SARS-CoV-2 S antibody concentrations at different time points after the initial two-vaccine series (Kasiwagi et al.^39)^ 2022) In patients treated with anti-TNF (A), there was no significant difference in antibody concentrations at 3-9 and 10-16 weeks, but antibody titers waned over time, and those at 20-28 weeks were significantly lower than those at 3-9 weeks. On the other hand, in both patients treated with non-anti-TNFα (B) and 5-ASA (C), antibody concentrations were sustained over time.

Kashiwagi et al.^39)^, another graduate student in our department, also examined 63 pediatric IBD patients who received two doses of the COVID-19 vaccine, focusing in particular on the impact of anti-TNFα antibody preparations on the ability to acquire and retain vaccine antibodies, the degree of short-term side effects after the first and second vaccine doses and the impact on IBD activity, and the booster effect after the third vaccine dose. All pediatric IBD patients were seroconverted, with no serious short-term adverse effects. Pediatric IBD patients on anti-TNFαhad significantly lower antibody titers than those on other medications at all measurement points (Figure 11). Furthermore, antibody titers waned over time with anti-TNFα and were significantly lower at 20-28 weeks than at 3-9 weeks after the two-vaccine series (Figure 12). In all ten patients, the third vaccination led to antibody concentrations significantly higher than those at the same time point after the second vaccination. Pediatric IBD patients on anti-TNFαneed to remain vigilant about COVID-19 even after two vaccinations, and a third vaccination may be considered.

**Figure 13:**
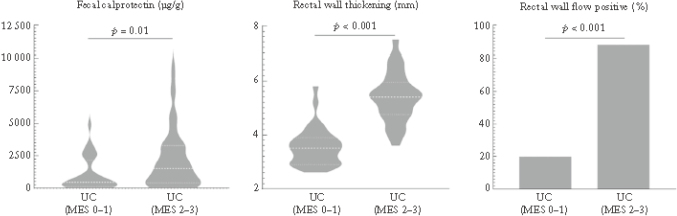
Change in fecal calprotectin and ultrasonic parameters in the Mayo endoscopic sub-score MES 0-1 and MES 2-3 groups (Jimbo et al.^40)^ 2023) Median value of fecal calprotectin, rectal wall thickening, and rectal wall flow in the two groups showed significant differences between both groups.

Transabdominal ultrasonography appears comparable to colonoscope for evaluating UC activity, but it has low accuracy in rectal evaluation. Jimbo et al.^40)^ compared the accuracy of transperineal ultrasonography for evaluating rectal activity to that of colonoscopy in pediatric UC cases. Fecal calprotectin values and transperineal ultrasonography and colonoscopy findings were compared prospectively in pediatric UC cases. Rectal wall thickening and rectal wall flow on power Doppler evaluated by transperineal ultrasonography were compared with colonoscopy findings and were also measured on transabdominal ultrasonography and assessed for the concordance rate of each finding. Median value of fecal calprotectin, rectal wall thickening, and rectal wall flow in the Mayo endoscopic sub- score 0-1 and 2-3 groups showed significant differences between both groups (Figure 13). The results showed that transperineal ultrasonography can evaluate rectal activity of UC with accuracy comparable to endoscopy. If accurate ultrasonic screening for the total colon can be performed by transperineal ultrasonography and transabdominal ultrasonography, repeated evaluation of short-term treatment response may be possible.

## Current status, issues and measures of transition for childhood-onset IBD patients

Transition in Japan is progressing along with legal arrangements, but the current situation is not necessarily smooth sailing. Rather, there are many issues that need to be resolved, and the situation is the same for childhood-onset IBD patients. In 2018, we conducted a questionnaire survey on the transition of childhood-onset IBD patients among adult gastroenterologists^41)^. The results showed that 76.4% of respondents “experienced some difficulty in accepting childhood-onset IBD patients referred by pediatricians specializing in gastroenterology,” and only 26.5% would accept them without hesitation. The reasons for this were found to be a lack of information provided by the pediatric side, communication between medical professionals, the patient's acceptance of non-anesthetized procedures, and the ability to handle problems at the time of consultation. The following year, in 2019, a similar questionnaire survey^42)^ was conducted among 48 members of the JSPGHAN. The results showed that 89.7% of respondents considered transfer to be an ideal form of medical care for childhood-onset IBD patients, while 58.6% said they had had a difficult experience. Furthermore, the fact that the provision of information from the pediatric department and the ability of the patient to identify the cause are the keys to successful transfer was consistent with the results of a questionnaire survey of adult gastroenterologists^41)^.

**Figure 14:**
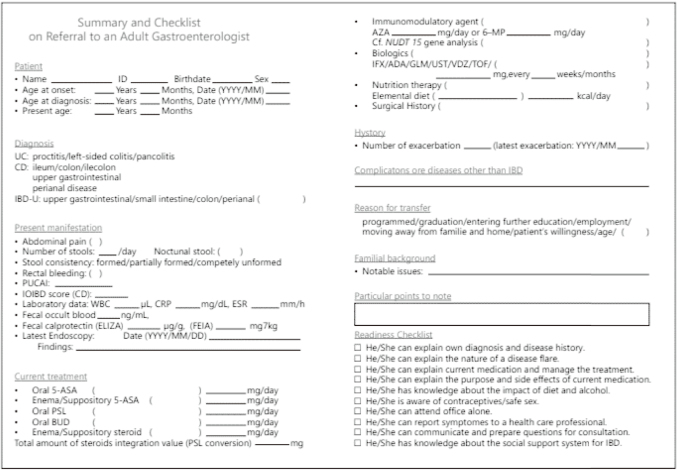
Proposed template for standardized medical summary with a checklist for referral PUCAI, Pediatric Ulcerative Colitis Activity Index; IOIBD, International Organization for Study of Inflammatory Bowel Disease.

Despite the above situation, measures are being steadily taken to advance the transition and transfer of childhood-onset IBD patients. One of these measures is the “Guidebook for supporting the independence of pediatric inflammatory bowel disease patients in the transition to adulthood [2017]”^43)^ created by the JSPGHAN and published on the society's website. This guidebook is useful as a basic tool for various professions participating in the transition to adulthood to recognize the goals and check the progress. In addition, the “Ulcerative Colitis and Crohn's Disease Diagnostic Criteria and Treatment Guidelines, 2022 Revised Edition”^44)^ contains an IBD transition checklist (Figure 14) that can be used to provide patient information that was cited by both adult gastroenterologists and pediatric gastroenterologists as factors for successful transfer in the above-mentioned questionnaire survey^41, 42)^. It is designed to compactly organize and record the essential information and is expected to be a support tool for promoting transition and transfer. One of the important points is to make patients and guardians aware of the concept of transition from prepuberty and to proceed with the program in a planned manner from an early stage. Ideally, a team medical system involving multiple professions would be formed to support the transition. Efforts must be tailored to the circumstances of each facility.

**Figure 15:**
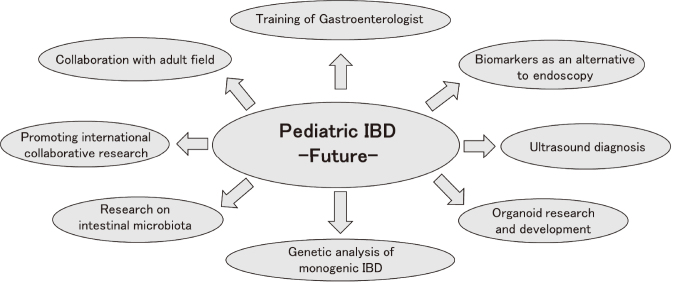
Pediatric inflammatory bowel disease - Future - (Shimizu^31)^ 2023) To accurately diagnose pediatric inflammatory bowel disease, provide appropriate treatment, and improve quality of life, it is important to take a comprehensive approach from clinical, research, and educational perspectives.

It is very important for pediatricians to properly diagnose and treat children with IBD, the number of whom is currently increasing rapidly. To achieve this, in the clinical field, it will be very important to use biomarkers and non-invasive diagnosis using ultrasound instead of endoscopy, and to collaborate with the adult field, in the research field to study intestinal bacteria and organoids, in the research field to promote international joint research on genetic analysis and therapeutic drugs for monogenic IBD, and in the education field to train pediatric gastroenterologists who can perform endoscopic examinations (Figure 15)^31)^.

## Conclusion

Although I have not been able to fully cover my journey as a professor of pediatrics at Juntendo University over the past 16 years, I believe I have been able to introduce some of my research progress. Of course, clinical work and education are important, but as an academic, one should place importance on research ability in one's path as a pediatrician. The past 16 years I have strongly experienced how this can lead to differentiation as a pediatrician. I would like to end my writing with the hope that the research mindset of young people will be nurtured without difficulty.

## Funding

No funding was received.

## Author contributions

The author contributed throughout the entire process of this paper.

## Conflicts of interest statement

The author declare that there are no conflicts of interest.

## References

[1] Matsui K, Takahashi K, Tanaka N, et al: Relationship between left ventricular deformation and early diastolic intraventricular pressure difference during rest and exercise. Juntendo Med J, 2016; 62: 26-33.

[2] Kobayashi M, Takahashi K, Yamada M, et al: Assessment of early diastolic intraventricular pressure gradient in the left ventricle among patients with repaired tetralogy of Fallot. Heart Vessels, 2017; 32: 1364-1374. 28634695 10.1007/s00380-017-1011-6

[3] Yamada M, Takahashi K, Kobayashi M, et al: Mechanism of left ventricular dysfunction assessed by layer-specific strain analysis in patients with repaired tetralogy of Fallot. Circ J, 2017; 81: 846-854. 28260735 10.1253/circj.CJ-16-1162

[4] Akimoto S, Fukunaga H, Akiya A, et al: Deep insight into cardiac dysfunction in children and young adults with Wolff-Parkinson-White syndrome using speckle tracking imaging. Heart Vessels, 2021; 36: 1712-1720. 34009415 10.1007/s00380-021-01848-5

[5] Yazaki K, Takahashi K, Shigemitsu S, et al: In-depth insight into the mechanisms of cardiac dysfunction in patients with childhood cancer after anthracycline treatment using layer-specific strain analysis. Circ J, 2018; 82: 715-723. 29187724 10.1253/circj.CJ-17-0874

[6] Shigemitsu S, Takahashi K, Yazaki K, et al: New insight into the intraventricular pressure gradient as a sensitive indicator of diastolic cardiac dysfunction in patients with childhood cancer after anthracycline therapy. Heart Vessels, 2019; 34: 992-1001. 30673819 10.1007/s00380-018-01332-7

[7] Ifuku M, Takahashi K, Hosono Y, et al: Left atrial dysfunction and stiffness in pediatric and adult patients with Type 1 diabetes mellitus assessed with speckle tracking echocardiography. Pediatr Diabetes, 2021; 22: 303-319. 33094524 10.1111/pedi.13141

[8] Akiya A, Takahashi K, Akimoto S, et al: Novel findings of early cardiac dysfunction in patients, with childhood-onset inflammatory bowel disease using layer-specific strain analysis. Inflamm Bowel Dis, 2023; 29: 1546-1554. 36971087 10.1093/ibd/izad031

[9] Umino D, Ohtomo Y, Hara S, et al: Serum indoxyl sulfate as an early marker for detecting chronic cyclosporine nephrotoxicity. Pediatr Int, 2010; 52: 257-261. 19761517 10.1111/j.1442-200X.2009.02961.x

[10] Endo A, Someya T, Nakagawa M, et al: Synergistic protective effects of mizoribine and angiotensin II receptor blockade on cyclosporine A nephropathy in rats. Peditar Res, 2014; 75: 38-44. 10.1038/pr.2013.169 24121426

[11] Sakuraya K, Endo A, Someya T, et al: The synergistic effect of mizoribine and a direct renin inhibitor, aliskiren, on unilateral ureteral obstruction induced renal fibrosis in rats. J Urol, 2014; 191: 1139-1146. 24140549 10.1016/j.juro.2013.10.053

[12] Mizutani, A, Endo A, Saito M, et al: Hydrogen-rich water reduced oxidative stress and renal fibrosis in rats with unilateral ureteral obstruction. Pediatr Res, 2022; 91: 1695-1702. 34365467 10.1038/s41390-021-01648-7

[13] Murano Y, Nishizaki N, Endo A, et al: Evaluation of kidney dysfunction and angiotensinogen as an early novel biomarker of intrauterine growth restricted offspring rats. Pediatr Res, 2015; 78: 678-682. 26270574 10.1038/pr.2015.153

[14] Nakagawa M, Nishizaki N, Endo A, et al: Impaired nephrogenesis in neonatal rats with oxygen-induced retinopathy. Pediatr Int, 2017; 59: 704-710. 28207964 10.1111/ped.13264

[15] Honjo A, Nakano N, Yamazaki S, et al: Pharmacologic inhibition of Notch signaling suppresses food antigen-induced mucosal mast cell hyperplasia. J allergy Clin Immunol, 2017; 139: 987-996. 27555456 10.1016/j.jaci.2016.05.046

[16] Yoneyama T, Nakano N, Hara M, et al: Notch signaling contributes to the establishment of sustained unresponsiveness to food allergens by oral immunotherapy. J Allergy Clin Immunol, 2021; 147: 1063-1076. 32717254 10.1016/j.jaci.2020.07.011

[17] Yamada H, Kaitani A, Izawa K, et al: Staphylococcus aureus δ-toxin present on skin promotes the development of food allergy in a murine model. Front Immunor, 2023; 14: 1173069. 10.3389/fimmu.2023.1173069 PMC10235538 37275864

[18] Ikeda N, Shoji H, Suganuma H, et al: Effect of insulin-like growth factor-I during the early postnatal period in intrauterine growth-restricted rats. Pediatr Int, 2016; 58: 353-358. 26635331 10.1111/ped.12855

[19] Tokita K, Shoji H, Arai Y, et al: Skeletal muscle insulin resistance in a novel fetal growth restriction model. Pediatr Rep, 2023; 15: 45-54. 36649006 10.3390/pediatric15010006 PMC9844385

[20] Santosa I, Shoji H, Arai Y, et al: Hepatic and skeletal muscle autophagy marker levels in rat models of prenatal and postnatal protein restriction. Nutrients, 2023; 15: 3058. 37447384 10.3390/nu15133058 PMC10346608

[21] Arai Y, Shoji H, Santosa I, et al: Effects of fetal growth restriction on postnatal gut microbiota in a rat model. J Pediatr Gastroenterol Nutr, 2023; 77: e42-e47. 37129884 10.1097/MPG.0000000000003805

[22] Ikeno M, Okumura A, Hayakawa M, et al: Fatty acid composition of the brain of intrauterine growth retardation rats and the effect of maternal docosahexaenoic acid enriched diet. Early Hum Dev, 2009; 85: 733-735. 19840892 10.1016/j.earlhumdev.2009.09.006

[23] Suganuma H, Arai Y, Kitamura Y, et al: Maternal docosahexaenoic acid-enriched diet prevents neonatal brain injury. Neuropathology, 2010; 30: 597-605. 20408962 10.1111/j.1440-1789.2010.01114.x

[24] Sakaguchi K, Koyanagi A, Kamachi F, et al: Breast-feeding regulates immune system development via transforming growth factor-β in mice pups. Pediatr Int, 2018; 60: 224-231. 29290091 10.1111/ped.13507

[25] Sato M, Kudo T, Arai N, et al: Evaluating small intestinal motility in a rat model of adolescent irritable bowel syndrome. Juntendo Med J, 2022; 68: 271-281. 10.14789/jmj.JMJ21-0050-OA PMC11250020 39021725

[26] Kyodo R, Kudo T, Ito N, et al: Modulation of intestinal motility in an adolescent rat model of irritable bowel syndrome. Digestion, 2024; 105: 99-106. 37963446 10.1159/000534732 PMC10994574

[27] Obayashi N, Ohtsuka Y, Hosoi K, et al: Comparison of gene expression between pediatric and adult gastric mucosa with helicobacter pylori infection. Helicobacter, 2019; 21: 114-123. 10.1111/hel.12245 26140656

[28] Miyata E, Kudo T, Ikuse T, et al: Eradication therapy for Helicobacter pylori infection based on the antimicrobial susceptibility test in children: A single-center study over 12 years. Helicobacter, 2021; 26: e12764. 33073418 10.1111/hel.12764

[29] Saito N, Suzuki M, Sakurai Y, et al: Genetic analysis of Japanese children with acute recurrent and chronic pancreatitis. J Pediatr Gastroenterol Nutr, 2016; 63: 431-436. 27409067 10.1097/MPG.0000000000001320

[30] Iso M, Suzuki M, Yanagi K, et al: The *CFTR* gene variants in Japanese children with idiopathic pancreatitis. Hum Genome Var, 2019; 6: 17. 30992994 10.1038/s41439-019-0049-7 PMC6459923

[31] Shimizu T: Pediatric inflammatory bowel disease -Past and Future-. Journal of the Japanese Society of Pediatrics, 2023; 127: 1155-1162. (in Japanese)

[32] Shimizu T, Suzuki M, Lee T, et al: Effects of n-3 polyunsaturated fatty acids and vitamin E on colonic mucosal leukotriene generation, lipid peroxidation, and microcirculation in rats with experimental colitis. Digestion, 2001; 63: 49-54. 11173900 10.1159/000051872

[33] Shimizu T, Kitamura T, Suzuki M, et al: Effects of alpha-linolenic acid on colonic secretion in rats with experimental colitis. J Gasroenterol, 2007; 42: 129-134. 10.1007/s00535-006-1998-4 17351801

[34] Shimizu T, Fujii T, Suzuki R, et al: Effects of highly purified eicosapentaenoic acid on erythrocyte fatty acid composition and leukocyte and colonic mucosa leukotriene B4 production in children with ulcerative colitis. J Pediatr Gastroenterol Nutr, 2003; 37: 581-585. 14581801 10.1097/00005176-200311000-00015

[35] Kudo T, Arai K, Uchida K, et al: Very early-onset inflammatory bowel disease in Japan: A nationwide survey. J Gastroenterol Hepatol, 2021; 36: 151-155. 32530546 10.1111/jgh.15146

[36] Morita M, Takeuchi I, Kato M, et al: Intestinal outcome of bone marrow transplantation for monogenic inflammatory bowel disease. Pediatr Int, 2022; 64: e14750. 33884705 10.1111/ped.14750

[37] Ito N, Kudo T, Eguchi H, et al: Attenuated expression of SLCO2A1 caused by DNA methylation in pediatric inflammatory bowel disease. Inflamm Bowel Dis, 2023; 29: 1920-1928. 37327083 10.1093/ibd/izad116

[38] Arai N, Kudo T, Tokita K, et al: Expression of oncogenic molecules in pediatric ulcerative colitis. Digestion, 2022; 103: 150-158. 34718239 10.1159/000519559 PMC8985031

[39] Kashiwagi K, Jinbo K, Suzuki M, et al: Impact of anti-TNFα treatment on the humoral response to BNT162b2 mRNA COVID-19 vaccine in pediatric inflammatory bowel disease patients, Vaccine (Basel), 2022; 10: 1618. 10.3390/vaccines10101618 PMC9611127 36298483

[40] Jimbo K, Hosoi K, Suzuki M, et al: Accuracy of transperineal ultrasonography for assessing rectal lesions in paediatric ulcerative colitis: a prospective study. J Crohns Colitis, 2023; 17: 1122-1127. 36920235 10.1093/ecco-jcc/jjad035

[41] Kumagai H, Kudo T, Uchida K, et al: Adult gastroenterologists' views on transitional care: Results from a survey. Pediatr Int, 2019; 61: 817-822. 31206932 10.1111/ped.13912

[42] Kumagai H, Kudo T, Uchida K, et al: Transitional care for inflammatory bowel disease: A survey of Japanese pediatric gastroenterologists. Pediatr Int, 20121; 63: 65-71. 10.1111/ped.14376 32621773

[43] Japanese Society of Pediatric Gastroenterology, Hepatology and Nutrition, “Guide to supporting the independence of pediatric patients with inflammatory bowel disease in the transition to adulthood [2017].” https://www.jspghan.org/guide/transition.html (Accessed Jul 1, 2024) (in Japanese)

[44] Ministry of Health, Laboure and Welfare Sciences Research Grant, Intractable Disease Policy Research Project, Research on Intractable Inflammatory Bowel Disorders (Hisamatsu Group), FY2022 Co-research Report: “Ulcerative colitis and Crohn's disease diagnostic criteria and treatment guidelines FY2022 revised edition.” http://www.ibdjapan.org/pdf/doc15.pdf (Accessed Jul 1, 2024) (in Japanese)

